# Preliminary Efficacy of a Gamified Mobile App for Promoting Self-Health Management Among Nurses in the Post-COVID Era: Single-Group Pre-Post Study

**DOI:** 10.2196/66262

**Published:** 2025-07-03

**Authors:** Shao Huan Hsu, Li Jung Lu, Pei Chin Chou

**Affiliations:** 1 College of Management Institute of Healthcare Information Management National Chung Cheng University Chiayi County Taiwan; 2 Department of Respiratory Care Chang Gung University of Science and Technology Chiayi County Taiwan

**Keywords:** gamification, mobile health, nurse wellness, self-health management, post-COVID, health care technology, behavioral intervention, digital health, health promotion, exercise adherence

## Abstract

**Background:**

The COVID-19 pandemic has significantly affected health care professionals, especially nurses, who have experienced elevated levels of stress, burnout, and physical health challenges. In the postpandemic era, supporting their well-being is crucial. Gamification, which is the application of game design elements in nongame contexts, has emerged as a promising strategy to promote engagement in health behaviors. This study explores the use of a gamified mobile app to support self-health management among nurses recovering from the COVID-19 experience.

**Objective:**

This study aimed to evaluate the preliminary efficacy of a gamified mobile app for promoting self-health management among nurses who experienced the COVID-19 pandemic. The study examined whether gamification could enhance engagement, improve physical health outcomes, and encourage sustainable behavior change.

**Methods:**

A single-arm pre-post intervention study was conducted using a user-centered design. The app was developed based on the Octalysis framework and goal-setting theory, incorporating personalized exercise prescriptions and health monitoring features. Nurses from a regional hospital in Hsinchu, Taiwan, participated in the 8-week intervention. Data were collected through interviews, pre- and postintervention surveys, and app usage analytics. Key outcomes included changes in step counts, BMI, and user engagement.

**Results:**

After the intervention, BMI classification improved significantly. The proportion of participants classified as obese decreased from 38.5% (90/234) to 13.7% (32/234), and the proportion of those classified as overweight increased from 24.8% (58/234) to 34.6% (81/234). Overall, the combined proportion of overweight or obese participants declined from 63.2% (148/234) to 48.3% (113/234), and that of participants with normal BMI increased from 18.4% (43/234) to 33.8% (79/234) (*χ*^2^_4_=29.98; *P*<.001). Octalysis tool results showed strong motivational engagement, with the highest scores in *development and accomplishment* (mean 7.29), *epic meaning and calling* (mean 7.05), and *empowerment of creativity and feedback* (mean 6.55).

**Conclusions:**

The gamified mobile app demonstrated promising efficacy in enhancing self-health management among nurses in the post-COVID era by increasing physical activity and improving BMI. Gamification elements, such as achievement, purpose, and feedback, were effective in sustaining engagement. Further studies are recommended to assess long-term outcomes and broader applicability.

## Introduction

### Background and Research Motivation

Since the COVID-19 outbreak in late 2019, health care systems have faced unprecedented stress. Nurses, particularly those on the frontlines, have experienced increased exposure to infection, mortality, psychological distress, and burnout [1-6]. Female nurses have been especially vulnerable [7-10]. Reports, such as *Who Cares Wins* by the United Nations and *ESG White Paper* by Taiwan’s Ministry of Health and Welfare, highlight the importance of workforce well-being within environmental, social, and governance (ESG) frameworks [11]. Despite these initiatives, nurse health continues to be threatened by excessive workload and chronic stress, compromising both individual well-being and the sustainability of health care systems.

To support the physical and mental health of health care workers, both self-management and external interventions are essential. Organizations like the World Health Organization (WHO) and the National Center for Chronic Disease Prevention and Health Promotion (NCCDPHP) recommend that adults engage in at least 150 minutes of moderate exercise weekly [12], but nurses often find it difficult due to shift work and fatigue [13]. As such, digital health platforms and mobile apps have gained popularity for providing accessible health management tools [14]. These include psychological screenings, mental health apps like BetterHelp and Calm [15], dietary tracking tools, and weight management apps that support goal-setting and outcomes [16-22].

Although digital health platforms offer structural support for health management, sustaining user engagement over time remains a major challenge. A key reason is the lack of intrinsic motivation and enjoyment, often resulting in user attrition [14,23]. This gap highlights the urgent need for more engaging and user-centered interventions.

### Introduction to the Literature Review

The American College of Sports Medicine advocates the FITT (frequency, intensity, time, and type) principle, a widely accepted method for exercise prescription. However, Burnet et al [24] argued that the FITT model lacks the element of “fun” and introduced the FITTF (frequency, intensity, time, type, and fun) model to improve user adherence. Yet, many health apps still fail to incorporate an enjoyable design, limiting user compliance. Gamification, which is the application of game elements to nongame contexts, has emerged as a promising strategy to enhance health behavior engagement. Initial gamified apps often lacked theoretical rigor, but more recent efforts have used motivational theories such as goal-setting, behavior change, and social influence. Alahäivälä and Oinas-Kukkonen [25] integrated gamification into health apps to boost self-management behaviors. Yang et al. [14] found that incorporating goal-based tasks and social interaction improved users’ health outcomes [26,27]. Competitive features like leaderboards can also enhance motivation, as shown by Landers et al [28,29], though some studies caution about the potential negative impacts of competition.

Chou [30] developed the Octalysis framework, combining game design, motivational psychology, and behavioral economics into 8 core drives. Although it is widely used in education and marketing [31-34], its application in health care remains limited, representing an area for further exploration. A systematic review by Xu et al [35] analyzed 50 studies on gamified health apps and reported mixed findings in terms of engagement and effectiveness. External motivators like rewards and rankings are common [36,37], but lasting behavior change often depends on internal motivation, such as personalized goal-setting. Landers et al [29] emphasized that well-designed gamification strategies can transform intrinsic desires into actionable goals [29]. While many apps employ external incentives, personal goals more effectively reflect users’ health needs and sustain engagement. Therefore, understanding how to balance achievement incentives and personal goal-setting is key to designing effective health management apps.

Despite growing interest in gamified health tools, few studies have examined their use among health care workers, particularly nurses, in the postpandemic context. Furthermore, the integration of the Octalysis framework with the FITTF model remains underexplored. There is a clear research gap in addressing how gamification can be tailored to health care professionals, whose unique work stressors and responsibilities differ from those of the general population.

This gap sets the stage for our study, which seeks to explore how gamification strategies, rooted in the Octalysis framework and guided by the FITTF principle, can be tailored for the postpandemic health management of nurses.

### Rationale for Selecting the Research Setting

This study was conducted at a branch of a medical center in Hsinchu City, Taiwan, which was selected for 3 main reasons. First, the hospital actively promotes employee health and participates in the Health Promoting Hospital initiative, providing a supportive environment for health interventions. Second, it has a stable and diverse nursing workforce, including various shift types and specialties, ensuring the inclusion of participants with different health needs. Third, the hospital is open to technological innovation and expressed a willingness to collaborate in the co-design and testing of the gamified app. These factors ensure practical feasibility, institutional support, and contextual relevance for evaluating the effectiveness of the intervention.

### Research Questions and Objectives

To address the identified gap, this study aims to design and evaluate a gamified mobile health management app specifically tailored for nurses. The app will incorporate the Octalysis framework and FITTF exercise prescription principles and will be iteratively refined through user feedback. The key research objectives are as follows: (1) to identify essential gamification features using the Octalysis framework, which effectively support nurses’ health needs after the COVID-19 pandemic, and (2) to develop and validate a FITTF-based exercise prescription that enhances nurses’ physical health and health management outcomes.

By focusing on health care workers, a frontline group severely affected by the pandemic, this study contributes to theoretical advancements in gamified health interventions and the practical development of scalable and sustainable self-management tools for health care professionals.

Based on previous literature, we hypothesized that gamification interventions would improve physical activity and BMI among health care workers in the postpandemic era and promote sustained engagement through motivational gamification features.

## Methods

### Overview

In light of the current literature, there has been limited research focused on developing gamified health management apps specifically designed for nurses in the postpandemic era. This study aims to address this gap by integrating the Octalysis gamification framework with the goal-setting theory to develop a gamified health management app tailored to nurses’ needs. The app’s design will draw on exercise prescriptions recommended by leading global health organizations and will be refined through in-depth interviews conducted with staff at a selected case hospital to ensure it meets user needs effectively.

### Study Design

This study adopted a single-group pre-post intervention design and was conducted over an 8-week period to evaluate the preliminary efficacy of a gamified mobile app in promoting self-health management among nurses in the post-COVID context.

### Participants and Recruitment

This study employed a purposive sampling strategy to recruit eligible participants. Anonymous online questionnaires were distributed via a secure web-based platform, and a total of 234 valid responses were included in the final analysis.

The inclusion criteria were as follows: (1) being a full-time employee of the participating hospital, (2) willingness to participate in the 8-week intervention program, and (3) ability to operate the mobile app independently.

The exclusion criteria were as follows: (1) medical conditions that limited or prohibited physical activity, (2) pregnancy, and (3) concurrent participation in other structured health or wellness programs during the study period.

### System Development Methodology and Design Philosophy

Two primary development methodologies were considered for this study: system development life cycle (SDLC) and prototyping.

#### SDLC Methodology

SDLC is a well-defined system development methodology consisting of several distinct phases, including system design, construction, testing, and delivery, which are all aimed at producing a high-quality system that meets or exceeds customer expectations [38].

#### Prototyping

Prototyping, on the other hand, is a system development methodology that emphasizes user feedback and rapid iteration. It involves creating a simplified initial version (prototype) of the system, which allows users to visualize and interact with the system’s design and features early on. The development team then refines the system based on user feedback, ultimately achieving a product that meets user needs. This approach is particularly suitable for projects with unclear or frequently changing requirements, as it shortens development cycles and enhances efficiency.

While SDLC offers a structured and comprehensive approach, it is time-consuming and costly, and often struggles to accommodate evolving requirements. Conversely, prototyping provides a quicker, more cost-effective solution that allows for iterative improvements based on user input. Given these advantages, this study opted for the prototyping method.

This study adopted a prototyping approach to system development, which emphasizes user-centered design, iterative refinement, and feedback integration. Prototyping allows users to interact with early-stage versions of the system, enabling developers to incorporate their feedback in real time and create a product that closely aligns with users’ actual needs. Compared to the more rigid and time-consuming SDLC methodology, prototyping is more flexible and efficient, making it suitable for projects with evolving requirements.

From a design perspective, this approach reflects a clear empowering orientation. By involving end users (nurses) in the design process through interviews and iterative feedback loops, the system fosters a sense of ownership and autonomy. This aligns with the principles of empowering technologies, which aim to enhance users’ self-efficacy and enable independent health management.

In addition to its empowering foundations, the system also incorporated hedonic elements in its gamification design. Drawing from the Octalysis framework, the final product included features, such as goal progression, achievement rewards, and friendly competition, which introduced playfulness, unpredictability, and social influence. These elements were designed to enhance enjoyment and intrinsic motivation, thereby supporting sustained user engagement in health behavior change.

Together, these complementary design philosophies, empowering users through co-design and engaging them through gamified, hedonic features, form the theoretical and practical backbone of the mobile app developed in this study. Moreover, although the system was not formally structured based on a cognitive behavioral therapy (CBT) framework, several features were aligned with CBT behavioral activation strategies. These included personalized goal-setting, self-monitoring dashboards, and timely performance feedback, which have been shown to support users in recognizing and modifying maladaptive behavior patterns. These strategies promote sustained engagement in physical activity and reflect core techniques commonly adopted in CBT-informed digital health interventions, such as those described in the work of Tudor-Sfetea et al [39]

### System Interviews

#### In-Depth Interviewing

In-depth interviewing, also known as qualitative interviewing, was categorized by Patton [40] into 3 forms: structured interview, unstandardized interview, and guided interview.

Structured interview is a quantitative research method commonly used in survey research. This approach standardizes interview questions and uses precise language to ensure that each respondent experiences the same questions and sequence, thus ensuring the reliability of responses.

Unstandardized interview is an open-ended interview method without standardized questions. The interviewer engages the respondent in a free-form discussion around a central theme, collecting data flexibly without the respondent’s awareness.

Guided interview is a semistructured interview method where the interviewer prepares a general outline of topics to be discussed. This approach allows for guided yet flexible conversations, enabling respondents to express their thoughts in a relaxed environment while ensuring that the discussion remains focused.

Given these advantages, this study employed guided interviews. The researcher prepared an interview outline and key questions in advance and made adjustments based on respondent feedback during the interviews. The complete interview guide is available in Multimedia Appendix 1. This approach provided rich, diverse data and deep insights into users’ real needs and challenges, informing the design of the gamified health management app.

Additionally, while the study did not implement a formal CBT protocol, several interview-informed design elements aligned with CBT principles. Specifically, the interviews helped identify behavioral barriers and health beliefs, which were subsequently addressed through in-app features such as goal-setting, self-monitoring, and activity prompts, which are key components associated with behavioral activation strategies in CBT. These elements encouraged users to recognize and alter unhelpful behavior patterns, supporting sustainable health management behavior.

The hospital’s Health Promotion Committee, led by the Deputy Director, has implemented several measures to enhance the health of employees, patients, and their families, creating a healthy hospital environment. The key measures include promoting mental support activities (providing psychological counseling and support to enhance employee mental health), implementing health checks and tracking (regular health checks with follow-up management of the results), conducting health promotion training (offering health knowledge and skills training to increase employee health literacy), enhancing health awareness (raising awareness and understanding of health management among employees through various forms of education and communication), and encouraging participation in health promotion activities (organizing and encouraging participation in health activities to foster a sense of engagement and motivation).

### Qualitative Interview Procedures

The initial interview outline was designed to address the challenges and needs nurses face in their daily work and health management, without being strictly based on any specific theoretical framework. Through open-ended interviews, the study aimed to uncover users’ real needs and difficulties. The interview results were used to establish the necessary functions for the app.

To inform the design of the gamified mobile health management app, a qualitative phase was conducted prior to system implementation. This phase involved semistructured interviews with frontline nurses using a guided interview protocol that explored challenges in self-health management and preferences for gamification features.

Participants were recruited through purposive sampling from among full-time nursing staff at the study hospital. All interviews were conducted in a private setting, audio recorded with participant consent, and transcribed verbatim. Thematic analysis was applied to the transcribed data using open coding techniques. Codes were iteratively grouped into categories, and overarching themes were identified through constant comparison, following established qualitative data analysis protocols.

Thematic analysis was conducted following the 6-phase framework by Braun and Clarke [41], including data familiarization, initial code generation, theme searching, theme review, definition, and reporting. Two researchers independently coded the interview transcripts manually and met regularly to compare interpretations, resolve discrepancies, and consolidate themes. Coding matrices and summary tables were used to organize and refine emerging categories. To enhance trustworthiness, peer debriefing was conducted and representative quotes were selected to support each theme.

### System Architecture Workflow

The gamified health management app developed in this study is designed to operate across various platforms. The app provides personalized exercise prescriptions based on user health data and allows users to store and query their health records. Nurses begin by registering a personal account and entering basic health data, such as age, height, weight, BMI, and fitness scores, via mobile devices. The system then evaluates and analyzes the data to generate a tailored exercise prescription, ensuring the appropriateness and effectiveness of the health plan.

### Demographic Data Collection

Prior to system implementation, participants completed a baseline self-administered questionnaire that collected demographic and health-related information. Items included age, gender, education level, occupation, regular exercise habits, and prior gaming behavior. The instrument consisted of structured multiple-choice and Likert-scale questions. Data were anonymized, and only fully completed responses were used for analysis.

### Gamification Design Features

Participants can engage in gamified challenges by completing the prescribed exercises, with the system offering rewards for successful completion to encourage ongoing engagement. The app was developed using the Octalysis framework, which informed the implementation of several core motivational drivers. Although visual screenshots were unavailable due to poststudy system access restrictions, the following features were integrated and aligned with key Octalysis dimensions: development and accomplishment (users tracked their daily steps via a visible progress bar and earned digital badges when achieving specific milestones), social influence and relatedness (a real-time leaderboard displayed peer rankings to foster a sense of friendly competition), empowerment of creativity and feedback (the app allowed avatar customization and delivered immediate feedback on user performance).

These features were designed to enhance intrinsic motivation and promote sustained behavior change in accordance with the Octalysis gamification model. In addition to aligning with core motivational drivers, these components also represent key hedonic (eg, badges and leaderboards) and empowering (eg, self-monitoring and avatar customization) gamification elements, consistent with the framework outlined by Lin et al [42].

Due to poststudy access restrictions, the system interface was not available for screenshot capture. Therefore, visual illustrations of the app’s design cannot be provided. However, detailed descriptions of each core component have been included to convey its structure and function clearly.

### Development Environment and Tools

The development environment and tools used in this study are outlined in Table 1.

**Table 1:** Development environment and tools.

Category	Environment/tool
Platform or system	Full platform (web version)
Development tools	Visual Studio Code and Firebase
Programming languages	HTML, CSS, JavaScript, Node.js, and SQL

### System Evaluation

This study developed a gamified health management app based on the Octalysis framework, which was used as the foundation for the app’s design. The efficacy of the system was assessed using the Octalysis tool, a specialized evaluation instrument that measures the app’s performance across 8 core drives of gamification. Additionally, a survey method was employed to gather user feedback and further evaluate the app’s efficacy.

Due to the anonymized and aggregate nature of the Octalysis tool output, individual participant-level responses were not stored. Therefore, SDs could not be computed for the core drive scores.

### Evaluation Methods

#### Octalysis Tool

The Octalysis tool was used for a comprehensive evaluation of the system. This professional tool assesses the efficacy of the gamified design in 8 core drives, including epic meaning and calling, development and accomplishment, and social influence. These core drives play a critical role in evaluating the system’s ability to motivate and engage users.

#### Survey Method

A survey was conducted using a Likert scale. The 10-point scale (ranging from “1” to “10”) was designed to collect user feedback on various aspects of the system’s functionality and user experience. The survey covered all core drives to ensure a balanced and comprehensive evaluation of the system’s overall efficacy.

### Evaluation Process

#### Survey Design and Distribution

The survey, structured around the 8 core drives of the Octalysis framework, was distributed to system users. The survey included multiple questions aimed at assessing user satisfaction and experience across different dimensions of the system.

#### Data Collection and Analysis

After collecting the survey responses, the data were analyzed using the Octalysis tool. The analysis involved calculating scores for each core drive, and an Octalysis diagram was generated to visually represent the system’s performance across these drives.

#### Result Interpretation and Improvement Recommendations

Based on the evaluation results, the system’s strengths and areas for improvement were identified. Specific recommendations were made to optimize the gamification design, enhance user experience, and improve the overall efficacy of health management within the system.

### Statistical Analysis Clarification

All statistical comparisons were conducted using SPSS version 25 (IBM Corp). To assess the significance of changes in BMI classifications before and after the 8-week intervention, a chi-square test was applied. The results indicated a statistically significant improvement in BMI distribution (*χ*^2^_4_=29.98; *P*<.001).

For Octalysis tool data, only aggregate average scores were available due to the tool’s design. Individual-level responses and SDs were not accessible, which prevented the use of inferential statistical tests, such as *t* tests or ANOVA, for gamification-related outcomes. As a result, these findings were reported descriptively.

Daily step data from the top 10 users on the leaderboard were also analyzed descriptively due to the limited subgroup sample size. Participants were categorized post hoc into “stable” and “unstable” engagement groups based on their activity patterns, which were further interpreted qualitatively through follow-up interviews.

Descriptive findings were transparently reported for all datasets where inferential testing was not feasible. Future studies are encouraged to incorporate larger samples and adopt analytical tools and designs, such as time-series models or regression-based approaches, that allow for more robust inferential analysis and causal inference.

### Ethical Considerations

This study was reviewed and approved by the Institutional Review Board of MacKay Memorial Hospital, Hsinchu Branch (approval number: 23MMHISO16e). Data were collected via an anonymous, web-based questionnaire embedded in a gamified mobile app. The survey did not collect any personal identifiers or sensitive health information. Participation was entirely voluntary. Before accessing the questionnaire, participants were presented with an electronic informed consent statement that explained the study’s purpose, procedures, data handling practices, and confidentiality assurances. Proceeding to complete the questionnaire was considered to indicate implied consent, in accordance with institutional ethical guidelines. All responses were anonymized, securely stored, and analyzed in aggregate. Only group-level results were reported to ensure the privacy and confidentiality of participants.

## Results

### Findings From In-Depth Interviews

Table 2 summarizes the key insights from the 3 interview sessions, which informed the design of the gamified mobile health management app.

During the interviews, users expressed needs that aligned with 4 core drives in the Octalysis framework: development and accomplishment, social influence and relatedness, unpredictability and curiosity, and loss and avoidance. These findings reflect key motivational drivers that informed the design of the intervention. These insights supported the selection of Octalysis as the theoretical foundation for the platform’s gamified feature design, ensuring both motivational alignment and sustained user engagement. This ensures that the app’s features align with nurses’ needs while leveraging gamification to enhance overall system efficacy.

**Table 2:** Interview results.

Session	Duration	Participants	Conclusion	Function requirements
1	2 hours	4	Main goal of the app: Weight control is a primary goal. Participants agreed that the app should focus on helping nurses maintain or achieve an ideal weight due to the nature of their busy and stressful work.	Pedometer, body data tracking, virtual island tour, leaderboard, and task and goal setting
2	2 hours	4	The app should provide personalized exercise prescriptions based on the user’s health status and must follow the FITT^a^ principle.	Exercise prescriptions, video tutorial links, and time-limited activities
3	2 hours	4	To enhance ongoing use, the app should include an achievement system where users can earn badges for reaching exercise goals.	Achievement system

^a^FITT: frequency, intensity, time, and type.

### Data Analysis

This study conducted an in-depth data analysis based on feedback from the questionnaire. To ensure the accuracy and completeness of the data, invalid responses were excluded, and valid responses were carefully categorized and summarized. Considering the specificity of this study, the Octalysis tool, which is designed to evaluate the system’s alignment with the Octalysis framework, was employed. The Octalysis tool, grounded in gamification theory, provides a comprehensive evaluation of the system’s performance in areas such as user experience, engagement, and motivational incentives. Quantitative analysis using the Octalysis tool was conducted to gain insights into the system’s strengths and areas for improvement.

### Questionnaire Response Rate and Demographic Analysis

This study used a purposive sampling strategy to recruit eligible participants. An anonymous online questionnaire was distributed via a secure web-based platform, resulting in 234 valid responses included in the final analysis. The analysis revealed that the majority of respondents were female (145/234, 62.0%), were aged between 31 and 50 years (123/234, 52.6%), held a university degree (137/234, 58.5%), and were engaged in nursing (143/234, 61.1%). Among the 234 respondents, 106 (45.3%) reported having a regular exercise habit, with most exercising less than 2 hours per week (79/106, 74.5%), and 128 (54.7%) did not have a regular exercise habit. Furthermore, of the 234 respondents, 17 (7.3%) reported playing sports-related games, with most of them playing for less than 5 hours per week (13/17, 76.5%), and the remaining 217 (92.7%) did not engage in such games. The data suggest that there is considerable room for improving exercise habits among the participants. The detailed findings of the demographic analysis are presented in Table 3.

**Table 3:** Participant demographics.

Variable category			Value (N=234), n (%)
**Gender**			
	Male	89 (38.0)	
	Female	145 (62.0)	
**Age (years)**			
	≤30	78 (33.3)	
	31-50	123 (52.6)	
	≥51	33 (14.1)	
**Education level**			
	High school/vocational	38 (16.2)	
	Associate degree	46 (19.7)	
	University	137 (58.5)	
	Graduate school	13 (5.6)	
**Occupation**			
	Health care related	143 (61.1)	
	Non–health care related	91 (38.9)	
**Exercise habit**			
	Yes	106 (45.3)	
	No	128 (54.7)	
**Sports-related game**			
	Yes	17 (7.3)	
	No	217 (92.7)	

### Octalysis Tool Analysis

Participants rated *development and accomplishment* and *epic meaning and calling* as the most engaging motivational dimensions, as shown in Table 4. These high scores likely reflect strong user interaction with gamification elements, such as the step tracking system, achievement badges, and progress bars, which were directly linked to these Octalysis core drives.

It is worth noting that a total of 198 valid responses were collected for the Octalysis survey, which was less than the responses for the demographic questionnaire. This discrepancy can be attributed to the different nature of the 2 questionnaires. The demographic survey was completed by participants before engaging with the gamified app, collecting general information such as background and occupation. Since it was administered early, participation was relatively high, leading to a larger number of responses. Conversely, the Octalysis assessment survey was completed after participants had engaged with the walking game, which may have led to lower response rates due to factors such as participant fatigue or decreased willingness to complete the survey after the intervention.

**Table 4:** Octalysis tool analysis.

Core drives	Average score^a^
Epic meaning and calling	7.05
Development and accomplishment	7.29
Empowerment of creativity and feedback	6.55
Ownership and possession	5.92
Social influence and relatedness	5.97
Scarcity and impatience	6.53
Curiosity and unpredictability	6.30
Loss and avoidance	6.30

^a^SDs could not be reported as the Octalysis tool provides only aggregate mean scores and does not retain individual-level response data.

#### Epic Meaning and Calling

Participants scored an average of 7.05 points in this dimension, indicating a relatively high level of engagement with the sense of mission and purpose within the walking game. This suggests that participants perceived the game as more than just a physical activity, attributing additional meaning and value to it, which enhanced their motivation and involvement.

#### Development and Accomplishment

In this dimension, participants scored an average of 7.29 points, reflecting positive feedback on the sense of achievement and personal progress within the game. This result suggests that the walking game successfully created a sense of accomplishment among participants, fostering a desire for personal growth and encouraging continued participation.

#### Empowerment of Creativity and Feedback

Participants scored an average of 6.55 points in this dimension, indicating a moderate response regarding exercise creativity and receiving immediate feedback within the game. This result implies that there is room for improvement in the game’s ability to stimulate creative thinking and provide timely and effective feedback. Future iterations of the gamified health management system should focus on enhancing these aspects to better meet participant expectations and needs.

#### Ownership and Possession

This dimension received the lowest average score of 5.92 points, indicating that participants felt a relatively low sense of ownership and possession within the game. This suggests that there is significant room for improvement in how the game fosters a sense of ownership and engagement with in-game items and related elements.

#### Social Influence and Relatedness

Participants scored an average of 5.97 points in this dimension, reflecting a relatively low need for social interaction and relatedness within the game. This finding suggests that the integration of social interaction elements in gamified tools should be approached with caution, ensuring that these elements align with the target audience’s preferences.

#### Scarcity and Impatience

In this dimension, participants scored an average of 6.53 points, indicating a moderate need for experiences of scarcity and urgency within the game. This suggests that incorporating elements that introduce challenge and a sense of time pressure could enhance participant engagement.

#### Curiosity and Unpredictability

Participants scored an average of 6.30 points in this dimension, indicating a moderate expectation for curiosity and unpredictability within the game. This highlights the importance of maintaining participants’ curiosity and excitement in future gamified tool designs.

#### Loss and Avoidance

Participants scored an average of 6.30 points in this dimension, indicating a moderate need to avoid losses within the game. This suggests that participants may have a desire to avoid failure and loss, providing a potential area for improvement in future iterations of the system.

Overall, the Octalysis tool analysis revealed the motivational structure of health care–related participants when engaging with the walking game. Participants responded most positively to the “epic meaning and calling” and “development and accomplishment” dimensions, while “ownership and possession” and “social influence and relatedness” received lower scores. These insights not only guide future improvements to gamified tools but also highlight the challenges of fostering social connections among health care professionals. These findings contribute to the precise adjustment of gamified elements to more effectively motivate and support health care workers in health management activities. Table 4 presents the average scores across the 8 core drives. Due to the design of the Octalysis tool, only aggregate mean scores were available, and individual-level response data could not be stored or accessed. As such, SDs could not be calculated for this dataset.

We agree that reporting SD is important for interpreting data variability and maintaining statistical rigor. In future studies, we recommend using engagement assessment tools that allow for the export of individual-level responses, which would enable more robust statistical analyses, including the calculation of SDs and inferential comparisons.

### Daily Step Data of the Top 10 Users on the Leaderboard

A key aspect of this study was to analyze the daily step data of the top 10 users on the leaderboard. According to the recommendations of the WHO and the American Heart Association (AHA), a daily step count of at least 10,000 steps is suggested to maintain basic health. This study used step count as the primary metric due to its ease of measurement, comprehensibility, and efficacy in reflecting daily activity levels [40]. Step count data are crucial indicators for evaluating individual physical activity levels, especially in research on health behaviors and lifestyle. The top 10 users on the leaderboard represented the most active participants in this study in terms of walking activity. Their step count trends revealed some interesting patterns that warrant further exploration. By conducting a detailed analysis of these users’ step data, we aimed to gain deeper insights into factors that may influence walking activity and provide valuable recommendations for encouraging healthier lifestyles and activity levels. In this section, we will present a detailed analysis and discussion of the daily step data of the top 10 users on the leaderboard, aiming to uncover trends and potential correlations.

Based on the variation in user step counts, these 10 users were categorized into 2 groups: stable and unstable. A more detailed analysis was conducted for each group to understand the reasons behind their step count changes and potential influencing factors.

#### Analysis of the Stable Group

User K825 consistently had a high step count from the beginning. On further interviews, it was revealed that this user had a pre-existing focus on health and exercise, along with a regular exercise routine.

Although there were some fluctuations in the step count of user L171, this user’s overall step count showed a gradual increase. This trend was attributed to the user’s growing awareness of the importance of exercise for health, leading to a deliberate effort to increase daily activity.

#### Analysis of the Unstable Group

The step count of user L821 was more volatile but showed a gradual increase, particularly in the final days, where there was a noticeable uptick. This change might be due to an increased health awareness or changes in the user’s lifestyle.

#### Analysis of Other Unstable Group Users

The step counts of these users were more erratic, with no clear trends. These users may require more support and encouragement to maintain a stable exercise routine.

As the activity period approached its conclusion, it appears that the users became aware that their rankings were unlikely to change, leading to a decrease in their motivation to continue increasing their step counts. In such scenarios, users may feel that further competition is unnecessary or may believe that additional steps will not significantly affect their rankings.

During the result analysis, interview findings revealed that users were motivated to exercise more when they saw the leading user putting in effort. This behavior indicates that users’ behavior changes were primarily driven by social influence rather than the Hawthorne effect. Social influence refers to the impact of others’ actions on an individual’s behavior, particularly when those individuals are seen as role models or leaders.

When users observed the leading user actively exercising, they felt encouraged to increase their own activity levels. This imitation behavior suggests that in group activities, individual behavior is often influenced by other group members. This underscores the importance of leveraging social influence when designing health management programs to motivate behavioral change.

This social influence can create a ripple effect within a group, where one person’s actions inspire others to follow suit, leading to an overall increase in group activity levels. Therefore, future research and interventions should capitalize on this by designing health behavior change programs that incorporate incentivizing social interactions and competitive elements.

### Pre- and Postintervention BMI Comparisons for All Users

To assess the changes in BMI among users before and after the fitness training program, this study conducted pre- and posttest comparisons of users’ BMI data, as summarized in Table 5, followed by further analysis.

Table 5 presents the distribution of participants’ BMI classifications before and after the 8-week intervention. The table includes both the number and percentage of participants in each BMI category (underweight, slightly underweight, normal, overweight, and obese), with notable shifts observed following the intervention. Specifically, the proportion of participants classified as obese significantly decreased from 38.5% (90/234) to 13.7% (32/234), while the proportion of those classified as overweight increased from 24.8% (58/234) to 34.6% (81/234). The combined percentage of overweight or obese participants declined from 63.2% (148/234) to 48.3% (113/234), accompanied by an increase in participants with normal BMI from 18.4% (43/234) to 33.8% (79/234). These changes were statistically significant, as confirmed by a chi-square test (*χ*^2^_4_=29.98; *P*<.001), indicating a meaningful improvement in participants’ weight status following the intervention.

Further analysis revealed that many individuals who were previously classified as obese successfully transitioned to the overweight or normal BMI category. Additionally, small changes were observed in the underweight category, with 1 participant shifting into the underweight category and 1 into the normal BMI category, possibly reflecting improved body awareness or behavior change. These findings support the efficacy of the gamified mobile app in promoting positive BMI outcomes among nurses in the post-COVID era.

**Table 5:** BMI classification before and after the intervention.

BMI category	Pretest^a^ (N=234), n (%)	Posttest^a^ (N=234), n (%)
Underweight	23 (9.8)	24 (10.3)
Slightly underweight	20 (8.5)	18 (7.7)
Normal	43 (18.4)	79 (33.8)
Overweight	58 (24.8)	81 (34.6)
Obese	90 (38.5)	32 (13.7)

^a^A chi-square test was conducted to compare pre- and postintervention distributions (*χ*^2^_4_=29.98; *P*<.001).

### Impact of the Gamified Health App on BMI Category Transitions

The data in Table 5 reveal significant shifts in BMI distribution following the fitness training intervention.

The number of users in the underweight category increased by 1, indicating that 1 user moved from the slightly underweight category to the underweight category. This may suggest that the user’s weight did not increase as expected after the intervention, possibly due to the absence of a dietary management feature in the study’s app.

The number of slightly underweight users decreased by 2, with 1 user moving to the underweight category and another successfully entering the normal BMI range, indicating a positive impact of the training on weight control for these users.

There was a significant increase in the number of users within the normal BMI range, rising from 43 in the pretest period to 79 in the posttest period. This increase was largely due to users from the overweight and obese categories moving toward a healthier range, suggesting that the majority of users who were previously overweight or slightly overweight achieved better weight control, reaching a normal BMI after the intervention.

Both the overweight and obese categories saw notable reductions in user numbers. The number of overweight users increased slightly from 58 to 81, primarily due to users from the obese category successfully reducing their weight and moving into the overweight range. However, the number of obese users decreased significantly from 90 to 32, indicating that a large portion of obese users were able to reduce their BMI to healthier levels through the training program.

## Discussion

### Principal Findings

This study evaluated the preliminary efficacy of a gamified mobile app designed to promote self-health management among nurses in the post-COVID context. The principal findings revealed that participants showed a significant improvement in BMI classification following the 8-week intervention, with a notable reduction in the proportion of individuals classified as obese. The app’s gamified features, developed using the Octalysis framework, successfully enhanced user engagement, with the highest motivational scores observed in the domains of *development and accomplishment* and *epic meaning and calling*. Additionally, qualitative interviews confirmed that nurses valued the app’s goal-setting, achievement rewards, and personalization features, which supported sustained participation. These results suggest that a well-designed gamified mobile health intervention can effectively promote physical activity and self-management behaviors in health care professionals.

### User Engagement Insights and Implications for Sustainable Health Interventions

This study’s participants primarily consisted of young, female health care professionals with university-level education, reflecting a demographic that makes up a significant portion of the nursing workforce. However, this concentration may limit the generalizability of findings to populations with different age distributions, gender ratios, or educational backgrounds. Age distribution showed that 33.3% (78/234) of participants were younger than 30 years, 52.6% (123/234) were between 31 and 50 years, and 14.1% (33/234) were older than 51 years. Interviews suggested that younger participants were more inclined to adopt new technologies and gamified apps, leading to higher engagement with the health management game. In contrast, older participants did not exhibit common motivations, indicating the need for tailored strategies across age groups. In terms of gender, female participants made up 62.0% (145/234) of the population and male participants made up 38.0% (89/234). Regarding education, 58.5% (137/234) had a university degree, 16.2% (38/234) had completed high school or vocational school, and 19.7% (46/234) held an associate degree. Only 5.6% (13/234) had a graduate degree. These findings suggest that women and individuals with higher educational attainment are more likely to engage in health management activities, which is crucial for future intervention design. Regarding exercise habits, 45.3% (106/234) of participants reported a regular exercise routine, with most of these exercising less than 2 hours per week. Only 7.3% (17/234) played exercise-related games, and 76.5% of these did so for fewer than 5 hours per week. This low participation in physical activity and gamified health apps underscores the need for enhanced strategies to increase engagement, especially among health care workers with limited leisure time due to heavy workloads. Activity tracking data revealed that step counts generally increased over time but declined near the end of the study. This pattern suggests that behavior was influenced by the perception of being monitored. Some users increased their activity due to perceived observation but relaxed their efforts once accountability diminished. This highlights the motivational role of structured timelines and perceived oversight in behavior change. Qualitative interviews emphasized the influence of peer leadership. For example, user K825 consistently demonstrated high activity levels and was seen as a motivational figure. This kind of role modeling aligns with the organizational health promotion theory. To succeed, institutions should foster a health-oriented culture by empowering peer leaders and providing the necessary support. Lastly, the long-term sustainability of health interventions depends on more than individual motivation. Continuous evaluation, user feedback, and supportive educational structures are needed. While identifying highly engaged individuals is a strong starting point, systemic support is essential for maintaining engagement and realizing sustained improvements in employee wellness.

### Comparison With Prior Work

This study contributes to the growing body of research on gamified health management apps, particularly in the context of health care workers, by addressing gaps and building on previous studies. Previous research has demonstrated the efficacy of gamification in various health-related interventions, such as promoting physical activity, enhancing adherence to healthy behaviors, and improving overall well-being [26,27]. However, much of the existing literature has focused on the general population or specific patient groups rather than health care workers, who face unique stressors and challenges in maintaining their health due to the demanding nature of their work [2,13].

Unlike earlier studies that predominantly examined the impact of gamification on a broader audience, this research specifically targeted health care professionals, particularly nurses in a post–COVID-19 environment. This focus allowed for a more nuanced understanding of how gamified interventions can be tailored to meet the specific needs of health care workers, addressing both physical and mental health challenges exacerbated by the pandemic. While previous studies have highlighted the potential of gamification to increase engagement and motivation [14,23], this study goes further by integrating the Octalysis framework and the FITTF principle, which adds a structured approach to designing gamified health interventions that are both engaging and effective.

Moreover, this study’s use of in-depth interviews and feedback loops to iteratively refine the app distinguishes it from prior research that often employed more static or one-size-fits-all gamification strategies. The focus on iterative design based on user feedback ensures that the app remains relevant and user-friendly, which has been identified as a critical factor in the success of health-related interventions [25].

In comparison to earlier studies, the findings of this study suggest that health care workers respond positively to gamified health management tools when these tools are specifically designed to align with their work environment and health needs. This contrasts with the more mixed results seen in broader population studies, where the efficacy of gamification can be less consistent due to the diversity of user backgrounds and motivations [35].

Overall, this research not only confirms the potential benefits of gamification in health management but also highlights the importance of context-specific design in enhancing the efficacy of these interventions for health care workers. It builds on prior work by offering a more targeted approach, contributing to a deeper understanding of how gamification can be effectively applied in high-stress, high-demand professional environments.

### Limitations

This section addresses the limitations encountered in this study and proposes potential avenues for improvement. Understanding these limitations is crucial for future researchers to consider the study’s findings more comprehensively and to guide further exploration in this field.

First, the participants in this study were selected from a specific health care institution, with a primary focus on health care workers within that institution. This focus allowed for an in-depth understanding of the particular needs and challenges within this specific work environment, enabling the provision of practical and tailored recommendations for the staff in this setting. Given that health care workers’ job characteristics, stressors, and health needs are significantly different from those of other professional groups, this targeted approach ensures the relevance and efficacy of the study’s conclusions.

However, this focus also limits the generalizability of the findings. In other types of health care institutions, such as large general hospitals, small clinics, or primary care facilities in remote areas, the work environment and staff needs may vary considerably. As a result, the study’s findings may not be fully applicable in these different contexts. Additionally, health care personnel in other functional areas, such as administrative management, logistical support, or technical roles, may experience different stressors and health needs compared to the clinical workers primarily considered in this study. Therefore, applying the study’s results to these other contexts should be done with caution. To address this limitation, future research should consider including participants from multiple institutions, settings, and functional roles to enhance the external validity and applicability of the findings.

Second, another limitation is the self-selection of participants, which introduces the potential for selection bias and may affect the representativeness of the study’s findings. Volunteers may be more motivated, health conscious, or interested in gamified interventions, which could lead to more favorable attitudes and outcomes during the study period. As such, the results may primarily reflect the characteristics of this specific subgroup rather than those of the broader population of health care workers. Furthermore, the sample was drawn from a single hospital, which may limit the generalizability of the findings to other clinical environments or to health care personnel in nonclinical roles. These factors should be carefully considered when interpreting the study outcomes.

Third, although 271 participants completed the baseline questionnaire, only 198 provided valid responses to the postintervention Octalysis assessment. This drop in the response rate may be attributed to participant fatigue, reduced motivation following the intervention period, or competing clinical duties. Such attrition may have introduced response bias and reduced the completeness and representativeness of the postintervention data. To mitigate this limitation in future studies, researchers should consider using shorter or more engaging follow-up instruments, sending scheduled reminders, or providing appropriate incentives to improve response rates and ensure data integrity.

Fourth, one of the most important limitations of this study is the use of a single-group pre-post design without a control or comparison group. While this approach allowed for the observation of changes in physical activity and BMI among the same participants, it inherently limited the ability to draw causal conclusions. Without a comparison group, it is not possible to rule out the influence of confounding factors or natural changes over time on the observed outcomes. External factors, such as seasonal variations, organizational initiatives, peer influence, or participants’ increased self-awareness, may have contributed to the improvements.

The absence of a control group was primarily due to practical and logistical constraints. The intervention was implemented in a real-world hospital setting where participants were also full-time clinical staff. Limited personnel availability, high clinical workload, and institutional restrictions on randomization made it infeasible to assign participants to a control or waitlist group without disrupting operations.

In addition, the sample consisted of voluntary participants recruited from a single institution. Individuals more interested in personal health or gamified technologies may have been more likely to participate, potentially leading to selection bias and limiting the generalizability of findings to the wider health care workforce.

Future studies should adopt randomized controlled trial (RCT) designs where possible. When full randomization is not feasible, alternative quasiexperimental designs, such as waitlist-control or matched cohort approaches, could provide a practical yet rigorous alternative to strengthen internal validity and causal inference while accommodating real-world constraints.

To address the limitations identified in this study, future research should consider adopting RCT designs to better establish causal relationships and minimize potential sources of bias. RCTs can provide more robust evidence of efficacy by controlling for external influences, accounting for natural changes over time, and improving the internal validity of the findings. Incorporating a control or comparison group would allow for a more accurate assessment of the intervention’s effects, and the use of stratified or random sampling methods would enhance the generalizability of the results across diverse populations and institutional settings. Although this study yielded valuable insights into the impact of a gamified intervention among health care workers in a specific institutional context, these findings should be applied with caution when extending to broader settings. Future studies are encouraged to explore long-term outcomes and assess intervention sustainability over time using more rigorous experimental designs.

### Recommendations

Based on the findings, hospitals should consider systematically incorporating gamified apps into employee wellness programs to improve nurses’ health behaviors. Management support, training programs, and continuous user feedback mechanisms should be established to sustain engagement. Future studies should adopt RCTs and broaden participant diversity to confirm the efficacy and generalizability of gamified health interventions in health care contexts.

### Future Directions and Follow-Up

In future research, we plan to adopt more rigorous experimental designs to strengthen the internal validity of the findings. Where feasible, an RCT design will be considered. Alternatively, quasiexperimental approaches, such as a waitlist-control group or matched cohort design, could offer a practical yet methodologically sound solution in clinical environments where full randomization is not feasible. These approaches would allow for clearer attribution of effects to the intervention while balancing real-world implementation constraints. Although this study focused primarily on physical health indicators, such as daily step counts and BMI, psychological dimensions, such as stress, anxiety, and emotional exhaustion, are equally important in understanding the holistic impact of health interventions. Future iterations of the app evaluation should incorporate validated tools, such as the Perceived Stress Scale (PSS) and Generalized Anxiety Disorder-7 (GAD-7), to assess mental well-being outcomes. While this study demonstrated short-term improvements in physical activity and BMI following the 8-week gamified intervention, long-term efficacy remains an important consideration. To address this, a follow-up study is planned to assess the sustainability of outcomes at 3 and 6 months after the intervention. This follow-up will involve reassessing participants’ daily step counts and BMI to determine whether behavior changes are maintained over time. Additional qualitative interviews will explore user experiences and identify factors influencing sustained engagement. These data will help inform refinements to the app and guide broader implementation strategies in future clinical settings.

### Conclusion

This study aimed to evaluate the preliminary efficacy of a gamified mobile app in promoting self-health management among nurses in the post-COVID era. The findings demonstrated statistically significant improvements in participants’ BMI classifications and strong motivational engagement driven by key gamification features designed using the Octalysis framework. The app successfully supported physical activity and goal-oriented behaviors through achievement systems, feedback mechanisms, and social influence. These results suggest that gamified digital health tools can serve as effective self-care interventions for health care professionals in high-stress settings. Further research is needed to assess the long-term effects and scalability of such interventions.

## Data Availability

The data sets generated and analyzed during this study are not publicly available due to institutional data protection policies and participant privacy considerations. However, deidentified data may be made available by the corresponding author upon reasonable request.

## Appendix

Multimedia Appendix 1 Draft interview outline.

## Abbreviations

CBT: cognitive behavioral therapy
FITT: frequency, intensity, time, and type
FITTF: frequency, intensity, time, type, and fun
RCT: randomized controlled trial
SDLC: system development life cycle
WHO: World Health Organization
