# Supplementary material for: Preliminary Efficacy of a Gamified Mobile App for Promoting Self-Health Management Among Nurses in the Post-COVID Era: Single-Group Pre-Post Study
Source: JMIR Serious Games. 2025 Jul 3;13:e66262. doi: 10.2196/66262 (PMC12278879; doi:10.2196/66262)
Supplement: Multimedia Appendix 1 [file games_v13i1e66262_app1.docx]

Multimedia Appendix 1

Interview Guide –Exploring Healthcare Professionals’ Needs and Preferences for a Gamified Mobile Health Application

This appendix provides the semi-structured interview guide used in the study to explore nurses’ health management behaviors and preferences regarding a gamified mobile health application.

Section A: Introduction and Consent

Thank you for participating in this interview.

The purpose of this discussion is to better understand your views on health management and your preferences for features in a gamified mobile application.

Your responses will remain confidential and used only for research purposes.

Do you consent to proceed with the interview?

## Section B: Health Management Behaviors

1. Can you describe your current health habits or routines (e.g., exercise, diet, stress management)?

2. What are the main challenges you face in maintaining your health while working as a nurse?

3. Have you used any health-related mobile apps before? If yes, which ones and how was your experience?

## Section C: Application Design Needs

4. What kind of features would you find helpful in a mobile app aimed at supporting your health?

5. How important is personalization in health recommendations for you (e.g., customized exercise plans)?

6. Would you find reminders, goal tracking, or feedback useful? Why or why not?

## Section D: Gamification Preferences

7. What are your thoughts on using game-like elements (such as points, badges, or leaderboards) in a health app?

8. Would social comparison or peer rankings motivate or discourage you?

9. How important is having a sense of progress or achievement in maintaining your engagement?

## Section E: Barriers and Suggestions

10. What barriers do you foresee in regularly using a health app?

11. What would make you more likely to continue using such an app over time?

12. Do you have any suggestions to make the app more engaging or supportive?

## Section F: Closing

Is there anything else you would like to share regarding health management or app design?

Thank you for your time and valuable insights.

Section A: Introduction and Consent

Thank you for participating in this interview.

The purpose of this discussion is to better understand your views on health management and your preferences for features in a gamified mobile application.

Your responses will remain confidential and used only for research purposes.

Do you consent to proceed with the interview?

## Section B: Health Management Behaviors

1. Can you describe your current health habits or routines (e.g., exercise, diet, stress management)?

2. What are the main challenges you face in maintaining your health while working as a nurse?

3. Have you used any health-related mobile apps before? If yes, which ones and how was your experience?

## Section C: Application Design Needs

4. What kind of features would you find helpful in a mobile app aimed at supporting your health?

5. How important is personalization in health recommendations for you (e.g., customized exercise plans)?

6. Would you find reminders, goal tracking, or feedback useful? Why or why not?

## Section D: Gamification Preferences

7. What are your thoughts on using game-like elements (such as points, badges, or leaderboards) in a health app?

8. Would social comparison or peer rankings motivate or discourage you?

9. How important is having a sense of progress or achievement in maintaining your engagement?

## Section E: Barriers and Suggestions

10. What barriers do you foresee in regularly using a health app?

11. What would make you more likely to continue using such an app over time?

12. Do you have any suggestions to make the app more engaging or supportive?

## Section F: Closing

Is there anything else you would like to share regarding health management or app design?

Thank you for your time and valuable insights.
